# Synthesis of C3-*epi*-virenose and anomerically activated derivatives

**DOI:** 10.1016/j.tetlet.2024.155041

**Published:** 2024-04

**Authors:** Liesa Röder, Sofia Torres Venegas, Klaus Wurst, Thomas Magauer

**Affiliations:** a Department of Organic Chemistry and Center for Molecular Biosciences, University of Innsbruck, Innrain 80–82, 6020 Innsbruck, Austria; b Department of General, Inorganic and Theoretical Chemistry, University of Innsbruck, 6020 Innsbruck, Austria

**Keywords:** Carbohydrate synthesis, Bioactive natural products, Anomeric activation, C3-*epi*-virenose

## Abstract

A 9-step synthetic route to a protected form of the C3-epimer of virenose from *D*-fucose is described. C3-*epi*-virenose is the carbohydrate unit of the bioactive polyketide elsamicin B and part of the carbohydrate unit of elsamicin A. The developed route enabled preparation of anomerically activated forms of this unique C6-deoxy sugar, including derivatives with 1-acetyl, 1-acetylthio, 1-trichloroacetimidate, 1-bromo, and 1-fluoro substituents.

Benzonaphthopyranone natural products are glycosidic polyketide antibiotics produced by various *Streptomyces* strains and include the gilvocarcins, chartreusin, chrymutasin, and hayumicins (Fig. 1) [1]. Notably, they are lactone-bridged biaryls with potent antitumor activities, making them promising candidates for the development of new anticancer therapeutics and antibiotics [1–3]. The *C*-glycosylated natural product chrysomycin A (**1**), a member of the gilvocarcins [4], has shown significant promise in cancer therapy by functioning as a novel inhibitor of the topoisomerase II enzyme [5]. Another noteworthy example is the *O*-aryl glycosylated polyketide antibiotic chartreusin (**2**), first isolated in 1953 from *Streptomyces chartreusis* [6].

Besides its interesting activity against certain Gram-positive organisms and mycobacteria [6], further studies revealed its significant antitumor activity in mice models against several cancer types, such as P388 and L1210 leukemia, B16 melanoma, and M5076 sarcoma [7,8]. Comparable antibacterial and antitumor activities were described for the 1986 isolated polyketides elsamicin A (**3**) (BMY-28090, elsamutricin) and elsamicin B (**4**), which are produced by the actinomycete strain J5907-21 [9,10]. In terms of mode of action, the elsamicin compound class demonstrates its cytotoxic effect by tightly associating with DNA, particularly targeting sequences rich in C and G bases. This interaction leads to the induction of strand scission and the formation of single-strand breaks when reducing agents are present. Notably, elsamicin A (**3**) stands out as one of the most potent inhibitors of topoisomerase II identified to date. Moreover, its binding to DNA appears to inhibit transcription [10–12].

Concerning the molecular structure of this class of natural product compounds, the DNA-intercalating unit is linked to a carbohydrate component. The incorporation of this carbohydrate unit is essential as it enhances water solubility, making these compounds potential candidates for drug development. Elsamicin A (**3**), in particular, has advanced to phase II clinical trials due to its high potency, attributed to increased water solubility of the amino sugar moiety [13].

The synthesis of methyl α-*D*-virenoside, the carbohydrate unit of chrysomycin A (**1**), was already accomplished in 1980 [14] and later modified in 2020 [4]. Surprisingly and to the best of our knowledge, synthetic access to the carbohydrate unit of elsamicin A (**3**) and elsamicin B (**4**), which is identified as a C3-epimer of C6-deoxy sugar virenose has not been reported. Here, we present a practical sequence for synthesizing this virenose C3-epimer, along with anomerically activated forms.

As depicted in Scheme 1, our initial steps involved peracetylation of *D*-fucose (**5**) using acetic anhydride and indium(III) triflate at ambient temperatures [15]. This resulted in the quantitative formation of tetraacetate **6**, which underwent bromination at the anomeric position when exposed to hydrobromic acid in acetic acid. The obtained fucosyl bromide is prone to hydrolysis and showed significant decomposition upon exposure to elevated temperatures or storage for more than one day. Therefore, the crude bromide was directly subjected to Zn-mediated elimination in the presence of sodium dihydrogen phosphate [16]. Under these conditions, the di-*O*-acetyl-*D*-fucal **7** was isolated in 38% yield over two steps.

To further advance in the synthesis, we deprotected both alcohols using potassium carbonate in methanol, resulting in a 68% yield of **8**. Fucal **8** was then protected employing standard conditions (BnBr, NaH) to afford the corresponding benzyl ether **9** in 88% yield. In hand with benzyl ether **9**, the stage was set for its selective oxidation to enone **10** [17]. To this end, a solution of **9** in acetonitrile in the presences of 4 Å molecular sieves was treated with Koser’s reagent [18,19]. Under these conditions, the enone **10** [20] was produced in 78% yield. The developed six-step sequence represents a robust access to enone **10**. The previously reported route to **10** by Bennett and coworkers proceeds via seven steps and involved a gold-catalyzed homopropargyl orthoester cyclization [20].

With enone **10** in hand, the stage was set for the introduction of the C3-methyl group through a 1,2-addition with methyllithium [21]. Performing the addition at −100 °C afforded the tertiary alcohol **11** as a single diastereomer in 94% yield. The high selectivity of this addition was rationalized by steric considerations. As illustrated in Scheme 1, the axial benzyl group hinders attack from the *Re* face in the half-chair conformation [21]. Subsequently, the tertiary alcohol **11** underwent dihydroxylation with catalytic amounts of osmium tetroxide (5 mol%) and stochiometric amounts of *N*-methyl-morpholine-*N*-oxide. The resulting triol was directly subjected to global acetyl protection using acetic anhydride and indium(III) triflate. The triacetate **12** was successfully isolated in 53% yield over two steps. The anomers (α:β ~ 2:1) were separable by high performance liquid chromatography. Their stereochemistry, specifically regarding the newly introduced acetate group at C2, was examined using 2D-NMR spectroscopy. The analysis of NOE correlations revealed the desired configuration for C2. Specifically, **α-12** exhibited a notable interaction between H_1_ and H_2_, which was absent in **β-12** (Fig. 2). In contrast, H_1_ in **ß-12** showed significant correlations with all other axial substituents, namely the methyl group at C3 and H_5_. Our findings were consistent with prior research indicating that dihydroxylation of glycals with osmium tetroxide predominantly yields the aldose form with *trans*-substituents at positions 2 and 3 [22,23]. The influence of substitution of the hydroxyl group at C3 was deemed negligible in this context [22,23].

To further modify the protecting group on C4, the benzyl group could be replaced with an acetate group through a two-step sequence. First, reductive benzyl deprotection with carried out with palladium on charcoal under hydrogen atmosphere. This was followed by acetyl protection using acetic anhydride and indium(III) triflate. The targeted tetraacetate **13** was obtained as anomeric mixture (α:β ~ 2:1) of the C3-epimer of protected virenose through this sequence in 97% yield over two steps.

After successfully establishing an efficient protocol for synthesizing tetraacetate **13**, our focus shifted to devising a suitable method for anomeric activation. Initially, we generated the corresponding anomeric bromide **15** by treating **13** with hydroboronic acid in acetic acid (Scheme 2). This bromide **15** was obtained as a single anomer in quantitative yield. Since the fucosyl bromide was found to be unstable upon exposure to silica gel and rapidly decomposed at elevated temperature, the crude material was directly utilized in subsequent transformations after isolation.

Furthermore, tetraacetate **13** served as the starting material for producing the thioacetate **14**. The latter was obtained in 61% yield as a ~ 1:0.30 anomeric mixture after treatment of **13** with trifluoromethanesulfonic acid and thioacetic acid in dichloromethane at 0 °C. For the selective removal of the anomeric acetyl group, the following sequence was applied. Treatment with hydrobromic acid gave the anomeric bromide **15** and addition of silver carbonate in an acetone–water mixture generated hemiacetal **16**. A direct method for converting tetraacetate **13** into **16** by treatment with hydrazine acetate did not went to full conversion in our hands. The hemiacetal **16**, which underwent rapid decomposition on silica gel, was employed without further purification for the synthesis of additional anomeric derivatives. For instance, glucopyranosyl trichloroacetimidates **18** were obtained in 49% yield (α:β ~ 6:1) after exposure of hemiacetal **16** to trichloroacetonitrile under basic conditions employing 1,8-diazabicyclo [5.4.0]undec-7-ene (DBU). Furthermore, introducing (diethylamino) sulfur trifluoride (DAST) to a solution of hemiacetal **16** in tetrahydrofuran at −40 °C yielded glycosyl fluorides **17**, which were obtained in a 61% yield (α:β ~ 5:1) over three steps. After separation of the anomers by flash column chromatography, the structure of **α-17** was validated via single-crystal X-ray analysis.

In conclusion, we developed the first synthesis of a protected form of C3-*epi*-virenose **12**. The stereocenter at C3 was set via a stereoselective 1,2-addition and the C3-hydroxy function was installed via dihydroxylation of the glycal. The developed strategy also provided access to five anomerically activated glycosides. These sugar donors feature 1-acetyl, 1-acetylthio, 1-trichloro-acetimidate, 1-bromo, and 1-fluoro substituents. Further studies towards the synthesis of glycosylated benzonaphtho-pyranones chartreusin (**2**), elsamicin A (**3**) and elsamicin B (**4**) are currently ongoing in our laboratories and will be reported in due course.

## Supplementary Material

**Appendix A. Supplementary data**

Supplementary data to this article can be found online at https://doi.org/10.1016/j.tetlet.2024.155041.

SI

## Figures and Tables

**Fig. 1:**
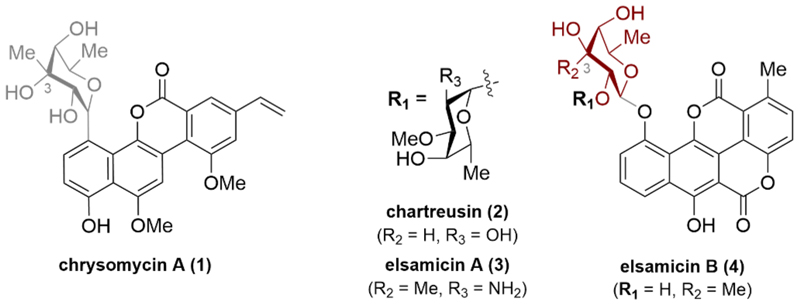
Selected structures of glycosidic polyketide antibiotics.

**Fig. 2:**
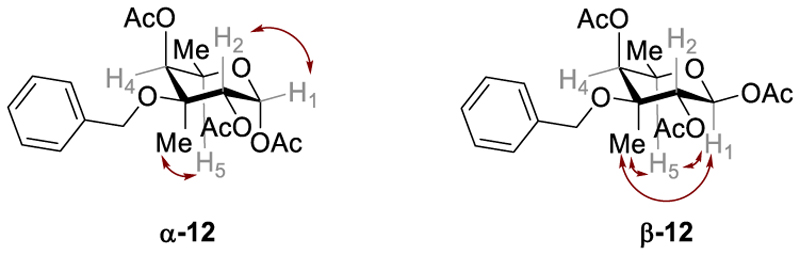
Selected NOE correlations for α-12 and β-12.

**Scheme 1:**
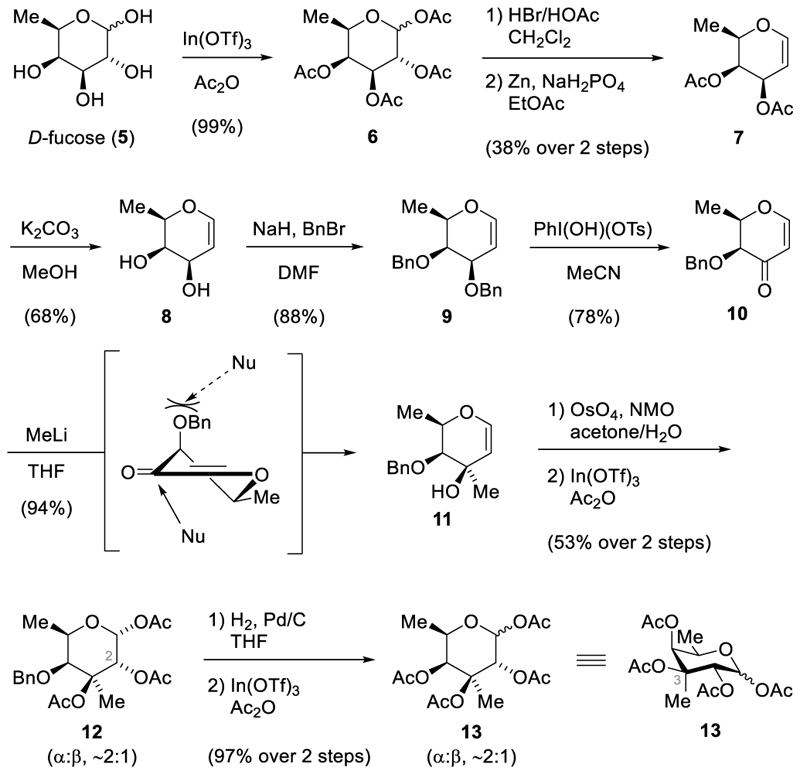
Synthesis of the C3-epimer of protected virenose 13.

**Scheme 2:**
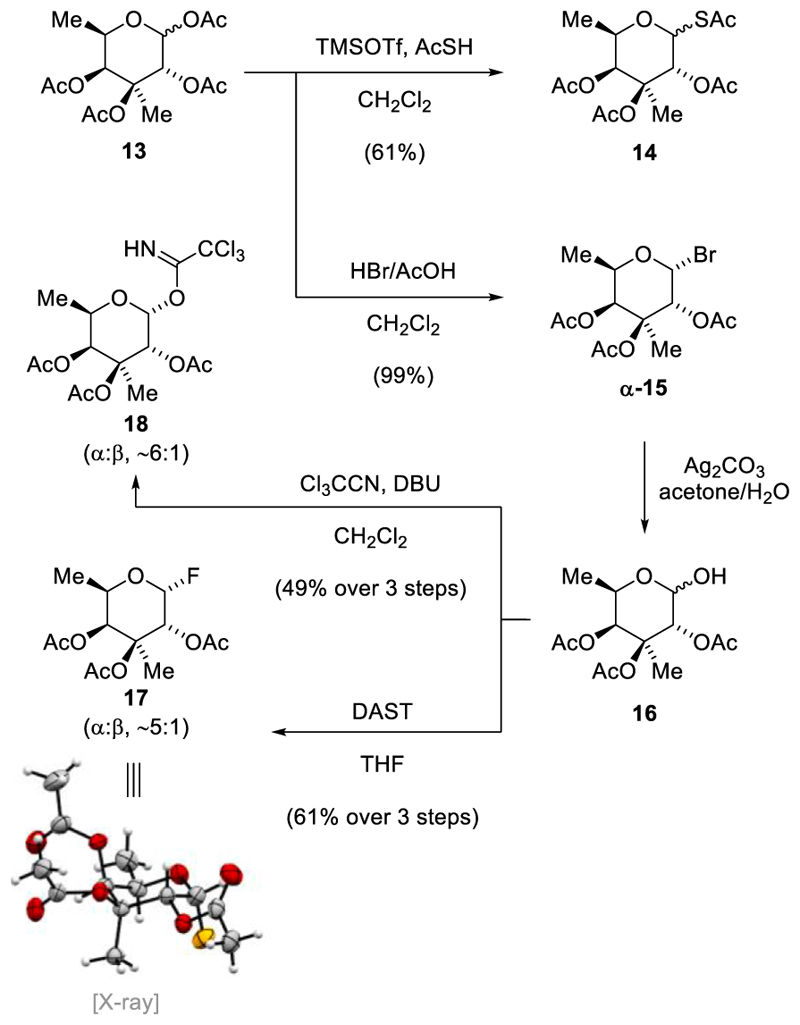
Synthesis of anomerically activated derivatives.

## Data Availability

Data will be made available on request.
