# Supplementary material for: Synthesis of C3-epi-virenose and anomerically activated derivatives
Source: Tetrahedron Lett. Author manuscript; Available in PMC 2024 Apr 25. (PMC7615872; doi:10.1016/j.tetlet.2024.155041)
Supplement: Supplementary file 1 [file EMS195553-supplement-SI.odt]

Supporting Information

Synthesis of C3-epi-Virenose and Anomerically Activated Derivatives

Liesa Rödera,†, Sofia Torres Venegas†, Klaus Wurstb, and Thomas Magauera*

a Department of Organic Chemistry and Center for Molecular Biosciences, University of Innsbruck, Innrain 80–82, 6020 Innsbruck, Austria.
b Department of General, Inorganic and Theoretical Chemistry, University of Innsbruck, 6020 Innsbruck, Austria.
*Email: thomas.magauer@uibk.ac.at

† L.R. and S.T.V. contributed equally to this work.

# Table of Contents

[1	   General Experimental Details	S3](#_Toc160469420)

[2	   Experimental Procedures	S6](#_Toc160469421)

[3	   NMR Spectra	S19](#_Toc160469422)

[4	   Crystallographic Data	S36](#_Toc160469423)

	

# 1	General Experimental Details

1.1.	General Working Methods

All reactions were carried out with magnetic stirring and, if moisture or air sensitive, under nitrogen or argon atmosphere using standard Schlenk techniques in oven-dried glassware (130 °C oven temperature). If required glassware was further dried under vacuum (0.1 mmHg) with a heat-gun at 650 °C. Air- and moisture-sensitive liquids were transferred via syringe or stainless-steel cannula through rubber septa. Solids were added under inert gas counter flow or were dissolved in appropriate solvents. External bath thermometers were used to record all reaction temperatures. Low temperature reactions were carried out in a Dewar vessel filled with acetone and dry ice (–‍78 °C) or equipped with an electronically regulated cryostat in acetone (­between –‍78 °C and 0 °C) or with distilled water and ice (0 °C). High temperature reactions were conducted in reaction vessels equipped with a reflux condenser or in a pressure tube using a heated silicon oil bath or a metal block. The reactions were magnetically stirred and monitored by NMR spectroscopy or analytical thin-layer chromatography (TLC), using aluminum plates percolated with silica gel (0.25 mm, 60 Å pore size, Merck) impregnated with a fluorescent indicator (254 nm). TLC plates were visualized by exposure to ultraviolet light (UV) at 254 nm, were stained by submersion in aqueous potassium permanganate solution (KMnO4) or ceric ammonium molybdate solution (CAM) and were developed by heating with a heat-gun. Flash column chromatography (FCC) was performed as described by Still[^1] employing silica gel (60 Å, 40–63 μm, Merck KGaA). The yields refer to chromatographically and spectroscopically (1H and 13C NMR) pure material, unless otherwise specified. 

1.2.	Materials

Tetrahydrofuran (THF) and diethyl ether (Et2O) were purchased from Merck as “anhydrous” and dried over molecular sieves (4 Å) prior to use. All other solvents were purchased from Acros Organics as ‘extra dry’ reagents. If required solvents were degassed by freeze-pump-thaw under vacuum (0.1 mmHg). All other reagents with a purity > 95% were obtained from commercial sources (Sigma Aldrich, TCI, Acros Organics, Alfa Aesar, Strem Chemicals, BLDpharm, Fisher Scientific, ABCR and others) and used without further purification unless otherwise stated. Solvents for extraction, crystallization and flash column chromatography were purchased in technical grade and distilled under reduced pressure prior to use. Lithium bromide was dried at 160 °C under vacuum (0.1 mmHg) for 12 h; the hot, dried solid was flame dried under vacuum (0.1 mmHg) for 4−5 minutes immediately prior to use. The molarity of n-butyllithium and t-butyllithium solutions was determined by titration against diphenylacetic acid as an indicator (average of three determinations).[^2]

1.3.	NMR Spectroscopy

NMR spectra (1H NMR, 13C NMR and 19F NMR) were recorded in deuterated chloroform (chloroform-d) or deuterated dimethyl sulfoxide (DMSO-d6) on a Bruker Avance Neo 400 MHz spectrometer, a Bruker Avance II 600 MHz spectrometer, or a Bruker 700 spectrometer at the Department of Organic Chemistry and Center for Molecular Biosciences, University of Innsbruck and are reported as follows: chemical shift δ in ppm (multiplicity, coupling constant J in Hz, number of protons) for 1H NMR spectra, chemical shift δ in ppm (multiplicity, coupling constant J in Hz) for 19F NMR spectra and chemical shift δ in ppm for 13C NMR spectra. Multiplicities are abbreviated as follows: s = singlet, d = doublet, t = triplet, q = quartet, p = quintet, br = broad, m = multiplet, or combinations thereof. In case of combined multiplicities, the multiplicity with the larger coupling constant is stated first. Expect for multiplets, the chemical shift of all signals, as well for centrosymmetric multiplets, is reported as the center of the resonance range. For 1H NMR the residual protic solvent peak served as internal reference (chloroform-d: 7.26 ppm, DMSO-d6: 2.50 ppm). For 13C NMR the central carbon resonance of chloroform-d (77.16 ppm) or DMSO-d6 (39.52 ppm) served as internal reference. NMR spectra were assigned using information ascertained from homonuclear correlation spectroscopy (COSY), heteronuclear multiple bond coherence (HMBC), heteronuclear single quantum coherence (HSQC) and nuclear Overhauser enhancement spectroscopy (NOESY) experiments. All raw fid files were processed, and the spectra analyzed using the software MestReNova 12.0.1 from Mestrelab Research S. L. 

1.4.	Mass Spectrometry

High resolution mass spectra (HRMS) as ESI-HRMS were recorded on a Thermo Scientific™ QExactive™ Orbitrap Mass Spectrometer at the Department of Organic Chemistry and Center for Molecular Biosciences, University of Innsbruck. DESI-HRMS as well as LTP-HRMS were recorded on a Thermo Scientific™ LTQ Orbitrap XL™ Hybrid Ion Trap-Orbitrap Mass Spectrometer equipped with a 3-in-1 ambient ionization interface[^3] at the Department of Organic Chemistry and Center for Molecular Biosciences, University of Innsbruck. 

1.5.	IR Spectroscopy

Infrared spectra (IR) were recorded from 4000 cm−1 to 450 cm−1 on a PerkinElmer Spectrum BX II FT-IR system. Samples were prepared as a neat film or a film by evaporation of a solution in chloroform-d. IR data in frequency of absorption (cm−1) is reported as follows: w = weak, m = medium, s = strong, br = broad or combinations thereof. 

1.6.	Optical Rotation

Optical rotation values were recorded on a Schmidt+Haensch UniPol L1000 Peltier polarimeter. 
The specific rotation is calculated as follows: 

Thereby, the wavelength λ is reported in nm and the measuring temperature ϕ in °C. α represents the recorded optical rotation at the apparatus, c the concentration of the analyte in 10 mg/mL and d the length of the cuvette in dm. Thus, the specific rotation is given in 10−1·deg·cm2·g−1. Usage of the sodium D line (λ = 589 nm) is indicated by D instead of the wavelength in nm. The respective concentration as well as the solvent is reported at the relevant section of the experimental section.

1.7.	X-Ray Diffraction Analysis

X-Ray diffraction analysis was carried out by Dr. Klaus Wurst at the Department of General, Inorganic and Theoretical Chemistry, University of Innsbruck. The data collections were performed on a Bruker D8 Quest diffractometer (Photon 100 detector) equipped with a microfocus source generator (Incoatec GmbH, Geesthacht, Germany) combined with multi-layer optics (monochromatized Mo Kα radiation, λ = 71.073 pm). The Bruker Apex III software was applied for the integration, scaling and multi-scan absorption correction of the data. The structure was solved with SHELXS (version 2013/1).[^4] Structure refinement (full-matrix least-squares against F²) with SHELXL (version 2014/7).[^5] All non-hydrogen atoms were refined anisotropically. The hydrogen atoms were placed in ideal geometry riding on their parent atoms. Further details are summarized in the tables at the different sections. Plotting of thermal ellipsoids in this document was carried out using MERCURY for Windows at 50% probability level.

# 2	Experimental Procedures

(3R,4S,5S,6R)-6-Methyltetrahydro-2H-pyran-2,3,4,5-tetrayl tetraacetate (6) 

Based on a slightly modified literature procedure,[^6] D-fucose (5) (5.00 g, 30.5 mmol, 1 equiv) was dissolved in acetic anhydride (31.5 mL) and indium(III) trifluoromethanesulfonate (51.4 mg, 91.4 μmol, 300 mmol%) was added at 23 °C. After five hours, the solvent was removed under reduced pressure and the crude reaction mixture was co-evaporated with toluene (2 × 20 mL). The residue was purified by flash column chromatography on silica gel (25% ethyl acetate in cyclohexane, grading to 30% ethyl acetate in cyclohexane) to yield acetyl fucopyranose 6 (10.0 g, 30.1 mmol, 99%, α:β ~ 4:1) as a colorless oil. The obtained analytical data were in full agreement with those reported in the literature.[^7]

(2R,3S,4R)-2-Methyl-3,4-dihydro-2H-pyran-3,4-diyl diacetate (7) 

Based on a slightly modified literature procedure,[^8] acetyl fucopyranose 6 (5.40 g, 16.2 mmol, 1 equiv) was dissolved in dichloromethane (15 mL). The solution was cooled to 0 °C and a solution of hydrogen bromide (30wt% in acetic acid, 13.2 mL, 73.1 mmol, 4.50 equiv) was added dropwise over a period of 30 min. Upon addition, the reaction mixture was allowed to warm up to 23 °C. After four hours, the mixture was cooled to 0 °C again and the cooled solution was slowly diluted with water (20 mL) and dichloromethane (15 mL). The layers were separated, and the aqueous layer was extracted with dichloromethane (3 × 50 mL). The combined organic layers were washed with a saturated aqueous solution of sodium bicarbonate (30 mL), followed by a saturated aqueous solution of sodium chloride (30 mL). The washed solution was dried over magnesium sulfate, the dried solution was filtered and the filtrate was concentrated under reduced pressure at 23 °C to afford a yellow syrup (assumed: 5.72 g, 16.2 mmol), which was used in the next step without further purification.

Based on a slightly modified literature procedure,[^9] a saturated aqueous solution of sodium dihydrogen orthophosphate (48 mL) was added to a solution of the crude fucosyl bromide (assumed: 5.72 g, 16.2 mmol, 1 equiv) in ethyl acetate (98 mL) at 23 °C. Afterwards, zinc dust (9.79 g, 150 mmol, 9.25 equiv) was added in small portions over 30 min at 23 °C. Upon addition, the reaction was stirred for further two hours at 23 °C before the layers were separated. The aqueous layer was extracted with ethyl acetate (3 × 40 mL) and the combined organic layers were washed with a saturated aqueous solution of sodium bicarbonate (50 mL) and water (30 mL). The washed solution was dried over magnesium sulfate, the dried solution was filtered and the filtrate was concentrated under reduced pressure. The residue was purified by flash column chromatography on silica gel (20% ethyl acetate in cyclohexane, grading to 25% ethyl acetate in cyclohexane) to yield fucal 7 (1.32 g, 6.15 mmol, 38% over two steps) as a colorless oil. The obtained analytical data were in full agreement with those reported in the literature.[^10]

(2R,3R,4R)-2-Methyl-3,4-dihydro-2H-pyran-3,4-diol (8) 

Based on a slightly modified literature procedure,15 potassium carbonate (510 mg, 3.69 mmol, 60.0 mol%) was added to a solution of fucal 7 (1.32 g, 6.15 mmol, 1 equiv) in methanol (43 mL) at 23 °C. After four hours, the solvent was removed under reduced pressure and the residue was purified by flash column chromatography on silica gel (40% ethyl acetate in cyclohexane, grading to 50% ethyl acetate in cyclohexane) to yield diol 8 (541 mg, 4.16 mmol, 68%) as a colorless solid. The obtained analytical data were in full agreement with those reported in the literature.[^11]

(2R,3S,4R)-3,4-Bis(benzyloxy)-2-methyl-3,4-dihydro-2H-pyran (9)

Based on a slightly modified literature procedure,[^12] sodium hydride (60% dispersion in mineral oil, 436 mg, 10.9 mmol, 3.00 equiv) was added to a solution of diol 8 (473 mg, 3.64 mmol, 1 equiv) in N,N-dimethylformamide (19 mL) at 0 °C. After 30 min, benzyl bromide (1.51 mL, 12.7 mmol, 3.50 equiv) was added. Upon addition, the reaction was allowed to warm up to 23 °C. After 14 h, the reaction was cooled to 0 °C and the cooled solution was diluted with water (10 mL) and diethyl ether (15 mL). The layers were separated and the aqueous layer was extracted with diethyl ether (3 × 30 mL). The combined organic layers were washed with a saturated aqueous solution of sodium chloride (20 mL). The washed solution was dried over magnesium sulfate, the dried solution was filtered and the filtrate was concentrated under reduced pressure. The crude product was purified by flash column chromatography on silica gel (5% ethyl acetate in cyclohexane, grading to 10% ethyl acetate in cyclohexane) to yield benzyl ether 9 (989 mg, 3.19 mmol, 88%) as a colorless oil.

TLC (10% ethyl acetate in hexanes): Rf = 0.26 (UV, CAM).

1H NMR (400 MHz, chloroform-d) δ 7.42 – 7.26 (m, 10H), 6.37 (dd, J = 6.3, 1.7 Hz, 1H), 4.97 (d, J = 12.0 Hz, 1H), 4.84 (ddd, J = 6.3, 2.3, 1.7 Hz, 1H), 4.77 – 4.68 (m, 2H), 4.63 (d, J = 12.2 Hz, 1H), 4.27 – 4.24 (m, 1H), 4.11 – 4.01 (m, 1H), 3.71 (dt, J = 3.8, 1.7 Hz, 1H), 1.29 (d, J = 6.6 Hz, 3H).

13C NMR (101 MHz, chloroform-d) δ 144.7, 138.7, 138.7, 128.5, 128.5, 128.4, 127.8, 127.7, 127.6, 99.6, 73.8, 73.3, 73.0, 72.3, 70.9, 16.7.

[α]D20 = +40.2 (c=1.00, CH2Cl2).

IR (Diamond-ATR, neat) max: 3030 (w), 2870 (w), 1644 (m), 1496 (w), 1453 (m), 1382 (w), 1346 (m), 1233 (s), 1171 (m), 1100 (vs), 1064 (vs), 1027 (s), 735 (s), 697 (vs) cm−1.

HRMS (ESI) calc. for C20H26O3N [M+NH4]+: 328.1907; found: 328.1914.

(2R,3S)-3-(Benzyloxy)-2-methyl-2,3-dihydro-4H-pyran-4-one (10)

Based on a slightly modified literature procedure,[^13] a suspension of benzyl ether 9 (565 mg, 1.82 mmol, 1 equiv) and grinded 4 Å molecular sieves (342 mg) in acetonitrile (37 mL) was stirred at 0 °C for 15 min. [Hydroxy(tosyloxy)iodo]benzene (Koser´s reagent) (856 mg, 2.18 mmol, 1.20 equiv) was added in one portion and the suspension was allowed to warm up to 23 °C. After 75 min, the mixture was filtered through a pad of Celite and the pad was rinsed with diethyl ether (25 mL). The filtrate was concentrated under reduced pressure and the residue was purified by flash column chromatography on silica gel (10% ethyl acetate in cyclohexane, grading to 20% ethyl acetate in cyclohexane) to furnish pyranone 10 (308 mg, 1.41 mmol, 78%) as a colorless oil. The obtained analytical data were in full agreement with those reported in the literature.[^14]

(2R,3S,4R)-3-(Benzyloxy)-2,4-dimethyl-3,4-dihydro-2H-pyran-4-ol (11)

Based on a slightly modified literature procedure,[^15] a solution of methyllithium (1.50 M in diethyl ether, 627 μL, 1.00 mmol, 1.50 equiv) was added dropwise to a solution of pyranone 10 (146 mg, 669 μmol, 1 equiv) in tetrahydrofuran (7.2 mL) at −100 °C. After 30 min, a saturated aqueous solution of ammonium chloride (7 mL) was added at −100 °C and the resulting biphasic reaction mixture was allowed to warm up to 23 °C. The layers were separated and the aqueous layer was extracted with diethyl ether (3 × 10 mL). The combined organic layers were washed with a saturated aqueous solution of sodium chloride (15 mL). The washed solution was dried over magnesium sulfate, the dried solution was filtered and the filtrate was concentrated under reduced pressure. The residue was purified by flash column chromatography on silica gel (20% ethyl acetate in cyclohexane) to afford fucal 11 (148 mg, 632 μmol, 94%) as a colorless oil.

TLC (15% ethyl acetate in toluene): Rf = 0.41 (CAM, ANIS).

1H NMR (400 MHz, chloroform-d) δ 7.42 – 7.29 (m, 5H), 6.28 (d, J = 6.3 Hz, 1H), 4.84 – 4.74 (m, 2H), 4.67 (dd, J = 6.3, 1.8 Hz, 1H), 4.12 – 4.07 (m, 1H), 3.28 (t, J = 1.5 Hz, 1H), 2.62 – 2.60 (m, 1H), 1.37 (s, 3H), 1.32 (d, J = 6.6 Hz, 3H).

13C NMR (101 MHz, chloroform-d) δ 143.2, 137.7, 128.7, 128.4, 128.2, 107.5, 81.5, 76.2, 72.2, 67.7, 29.0, 17.3.

[α]D20 = +7.9 (c=0.98, CH2Cl2).

IR (Diamond-ATR, neat) max: 3309 (br), 2899 (w), 1641 (s), 1503 (w), 1450 (m), 1357 (s), 1222 (vs), 1175 (s), 1094 (vs), 1080 (vs), 1050 (vs), 1045 (vs), 1040 (s), 793 (m), 731 (vs), 697 (s) cm−1.

HRMS (ESI) calc. for C14H18NaO3 [M+Na]+: 257.1148; found: 257.1144.

(3R,4S,5S,6R)-5-(Benzyloxy)-4,6-dimethyltetrahydro-2H-pyran-2,3,4-triyl triacetate (12)

A solution of osmium tetroxide (2.50wt% in tert-butyl alcohol, 65.4 μL, 31.6 μmol, 5.00 mol%) was added dropwise to a solution of 4-methylmorpholine 4-oxide (81.4 mg, 695 μmol, 1.10 equiv) and fucal 11 (148 mg, 632 μmol, 1 equiv) in acetone (6 mL) and water (1.5 mL) at 0 °C. Upon addition, the reaction was allowed to warm up to 23 °C. After 48 h, a saturated aqueous solution of sodium bicarbonate (5 mL) was added and the aqueous layer was extracted with ethyl acetate (3 × 6 mL). The combined organic layers were washed with a saturated aqueous solution of sodium chloride (10 mL). The washed solution was dried over magnesium sulfate, the dried solution was filtered and the filtrate was concentrated under reduced pressure to yield the triol (assumed: 169 mg, 632 μmol) which was dried azeotropically with toluene and used in the next step without further purification.

The unpurified triol (assumed: 169 mg, 632 μmol, 1 equiv) was dissolved in acetic anhydride (596 μL, 6.32 mmol, 10.0 equiv) and indium(III) trifluoromethanesulfonate (10.7 mg, 19.0 μmol, 3.00 mol%) was added at 23 °C. After three hours, volatile compounds were removed under reduced pressure and the residue was purified by flash column chromatography on silica gel (10% ethyl acetate in cyclohexane, grading to 30% ethyl acetate in cyclohexane) to yield an anomeric mixture of triacetate 12 (132 mg, 335 μmol, 53% over two steps, α:β ~ 2:1). An aliquot of the obtained anomeric mixture was subjected to semi-preparative HPLC (10% grading to 40% ethyl acetate in n-hexane in 40 min) to yield the separated α-anomer as a colorless amorphous solid and the β-anomer as a colorless oil for further analytics. 

α-12 

TLC (50% ethyl acetate in cyclohexane): Rf = 0.51 (CAM).

1H NMR (400 MHz, chloroform-d) δ 7.36 – 7.26 (m, 5H, Harom (OBn)), 6.30 (d, J = 4.5 Hz, 1H, 1-H), 5.50 (d, J = 4.4 Hz, 1H, 2-H), 4.72 (d, J = 11.7 Hz, 1H, CH2 (OBn)), 4.61 (d, J = 11.7 Hz, 1H, CH2 (OBn)), 4.37 (brs, 1H, 4-H), 4.19 (q, J = 6.5 Hz, 1H, 5-H), 2.12 (s, 3H, OAc), 2.06 (s, 3H, OAc), 1.83 – 1.80 (m, 6H, OAc and 6-Me), 1.28 (d, J = 6.5 Hz, 3H, 7-Me).

13C NMR (101 MHz, chloroform-d) δ 171.0, 169.9, 169.3, 138.4, 128.5, 127.7, 127.3, 90.4, 82.1, 82.0, 75.9, 69.6, 68.1, 22.3, 21.2, 20.8, 17.9, 17.5.

[α]D20 = +128.7 (c=0.20, CH2Cl2).

IR (Diamond-ATR, neat) max: 2990 (w), 2938 (w), 1755 (s), 1497 (w), 1456 (w), 1371 (m), 1235 (s), 1148 (m), 1070 (s), 1012 (m), 924 (w), 866 (w), 821 (w), 736 (w), 703 (w), 600 (w), 518 (w), 489 (w), 447 (w), 421 (w) cm−1.

HRMS (ESI) calc. for C20H26NaO8 [M+Na]+: 417.1520; found: 417.1508.

β-12 

TLC (50% ethyl acetate in cyclohexane): Rf = 0.50 (CAM).

1H NMR (400 MHz, chloroform-d) δ 7.38 – 7.26 (m, 5H, Harom (OBn)), 5.73 (d, J = 8.5 Hz, 1H, 1-H), 5.51 (d, J = 8.5 Hz, 1H, 2-H), 4.72 (d, J = 11.8 Hz, 1H, CH2 (OBn)), 4.61 (d, J = 11.9 Hz, 1H, CH2 (OBn)), 4.28 (brs, 1H, 4-H), 3.97 – 3.91 (m, 1H, 5-H), 2.10 – 2.08 (m, 6H, 2 x OAc), 1.81 (s, 3H, OAc), 1.67 (s, 3H, 6-Me), 1.30 (d, J = 6.4 Hz, 3H, 7-Me).

13C NMR (101 MHz, chloroform-d) δ 171.0, 169.8, 169.3, 138.4, 128.4, 127.6, 127.4, 91.2, 83.1, 81.7, 75.8, 71.3, 71.1, 22.1, 21.1, 21.0, 17.6, 17.3.

[α]D20 = +52.6 (c=0.15, CH2Cl2).

IR (Diamond-ATR, neat) max: 2990 (w), 2938 (w), 2880 (w), 1757 (s), 1497 (w), 1456 (w), 1371 (m), 1229 (s), 1171 (w), 1142 (w), 1052 (s), 918 (w), 852 (w), 740 (w), 715 (w), 604 (w), 567 (w), 503 (w), 443 (w) cm−1.

HRMS (ESI) calc. for C20H26NaO8 [M+Na]+: 417.1520; found: 417.1510.

(3R,4S,5S,6R)-4,6-Dimethyltetrahydro-2H-pyran-2,3,4,5-tetrayl tetraacetate (13)

Palladium on carbon (30wt% palladium, 11.9 mg, 33.5 μmol, 10.0 mol%) was added to a solution of triacetate 12 (132 mg, 335 μmol, 1 equiv) in tetrahydrofuran (3 mL) at 23 °C. An atmosphere of hydrogen was maintained by sparging with a stream of pure hydrogen gas through a stainless-steel needle for five minutes and vigorous stirring of the suspension was then continued under hydrogen atmosphere at 23 °C. After 18 h, the reaction mixture was filtered through a pad of Celite and the pad was rinsed with ethyl acetate (8 mL). The filtrate was concentrated under reduced pressure to afford the secondary alcohol (assumed: 102 mg, 335 μmol) as a colorless oil, which was used in the next step without further purification.

The unpurified secondary alcohol (assumed: 102 mg, 335 μmol, 1 equiv) was dissolved in acetic anhydride (316 μL, 3.35 mmol, 10.0 equiv) and indium(III) trifluoromethanesulfonate (2.83 mg, 5.03 μmol, 2.00 mol%) was added at 23 °C. After six hours, volatile compounds were removed under reduced pressure and the residue was purified by flash column chromatography on silica gel (25% ethyl acetate in cyclohexane) to yield an anomeric mixture of tetraacetate 13 (112 mg, 323 μmol, 97% over two steps, α:β ~ 2:1) as a colorless oil.

TLC (50% ethyl acetate in cyclohexane): Rf = 0.40 (CAM).

1H NMR (400 MHz, chloroform-d) δ 6.29 (d, J = 4.5 Hz, 1H)*, 5.78 – 5.73 (m, 1.54H)*, 5.70 – 5.68 (m, 0.54H), 5.41 – 5.37 (m, 1.54H)*, 4.29 – 4.22 (m, 1H)*, 4.05 – 3.99 (m, 0.54H), 2.16 (s, 1.62H), 2.15 (s, 3H)*, 2.13 (s, 3H)*, 2.10 (s, 1.62H), 2.09 (s, 1.62H), 2.08 (s, 3H)*, 1.93 (s, 4.62H)*, 1.87 (s, 3H)*, 1.75 (s, 1.62H), 1.20 (d, J = 6.4 Hz, 1.62H), 1.15 (d, J = 6.5 Hz, 3H)*.

Signals, which belong to the major α-anomer, are indicated with an asterisk.*

13C NMR (101 MHz, chloroform-d) δ 169.9*, 169.8, 169.8*, 169.5, 169.4*, 169.4, 169.1*, 91.1, 90.0*, 81.1, 79.9*, 74.0*, 73.6, 71.1, 69.8, 69.4*, 66.8*, 22.0*, 21.9, 21.2*, 21.0, 20.9, 20.8*, 20.7*, 17.8*, 17.4, 17.0*, 16.9.

IR (Diamond-ATR, neat) max: 2990 (w), 2926 (w), 1748 (s), 1439 (w), 1371 (m), 1214 (s), 1144 (m), 1064 (s), 1012 (m), 936 (m), 823 (w), 738 (w), 676 (w), 643 (w), 592 (w), 487 (w), 456 (w) cm−1.

HRMS (ESI) calc. for C15H22NaO9 [M+Na]+: 369.1156; found: 369.1148.

(3R,4S,5S,6R)-2-(Acetylthio)-4,6-dimethyltetrahydro-2H-pyran-3,4,5-triyl triacetate (14) 

Thioacetic acid (4.12 μL, 57.8 μmol, 2.00 equiv) was added to a solution of tetraacetate 13 (10.0 mg, 28.9 μmol, 1 equiv) in dichlormethane (100 μL) at 23 °C. The solution was cooled to 0 °C and after 15 min, trimethylsilyl triflate (10.6 μL, 57.8 μmol, 2.00 equiv) was added dropwise at 0 °C. After one and a half hour, the ice bath was removed and a saturated aqueous solution of sodium bicarbonate (500 μL) was added. The layers were separated and the aqueous layer was extracted with dichloromethane (3 × 1 mL). The combined organic layers were washed with saturated aqueous solution of sodium chloride (2 mL). The washed solution was dried over magnesium sulfate, the dried solution was filtered and the filtrate was concentrated under reduced pressure. The crude product was purified by flash column chromatography on silica gel (15% ethyl acetate in cyclohexane, grading to 25% ethyl acetate in cyclohexane) to afford an anomeric mixture of thioacetate 14 (6.40 mg, 17.7 μmol, 61%, anomeric ratio ~ 1:0.30) as a colorless oil.

TLC (50% ethyl acetate in cyclohexane): Rf = 0.43 (UV, CAM).

1H NMR (400 MHz, chloroform-d) δ 6.22 (d, J = 6.3 Hz, 0.3H), 5.71 – 5.69 (m, 1H)*, 5.68 – 5.67 (m, 0.3H), 5.60 (d, J = 6.4 Hz, 0.3H), 5.40 – 5.32 (m, 2H)*, 4.04 – 3.98 (m, 1.3H)*, 2.39 (s, 0.9H), 2.37 (s, 3H)*, 2.15 (s, 3H)*, 2.14 (s, 0.9H), 2.09 – 2.07 (m, 3.9H)*, 1.94 (s, 0.9H), 1.92 (s, 3H)*, 1.80 (s, 3H)*, 1.76 (s, 0.9H), 1.16 (d, J = 6.4 Hz, 3H)*, 1.14 (d, J = 6.5 Hz, 0.9H).

Signals, which belong to the major anomer, are indicated with an asterisk.*

13C NMR (101 MHz, chloroform-d) δ 193.0*, 192.5, 169.9*, 169.8, 169.6, 169.6*, 169.4*, 169.3, 81.3*, 80.1, 79.5, 79.1*, 74.0*, 73.7, 72.6*, 69.7, 69.6*, 67.8, 31.5, 31.0*, 22.0, 21.8*, 21.0*, 20.9, 20.8*, 20.7, 17.5, 17.2*, 17.1, 16.8*.

Signals, which belong to the major anomer, are indicated with an asterisk.*

IR (Diamond-ATR, neat) max: 2990 (w), 2942 (w), 1748 (s), 1707 (m), 1439 (w), 1371 (m), 1217 (s), 1126 (m), 1062 (s), 1019 (m), 953 (m), 920 (m), 889 (w), 839 (w), 788 (w), 732 (m), 621 (m), 557 (w), 509 (w), 478 (w), 445 (w) cm−1.

HRMS (ESI) calc. for C15H22NaO8S [M+Na]+: 385.0928; found: 385.0919.

(2R,3R,4S,5S,6R)-2-Bromo-4,6-dimethyltetrahydro-2H-pyran-3,4,5-triyl triacetate (15)

Tetraacetate 13 (20.0 mg, 57.7 μmol, 1 equiv) was dissolved in dichloromethane (300 μL). The solution was cooled to 0 °C and a solution of hydrobromic acid (30wt% in acetic acid, 47.0 μL, 260 μmol, 4.50 equiv) was added dropwise over a period of five minutes. Upon addition, the reaction mixture was allowed to warm up to 23 °C. After five hours, the mixture was cooled to 0 °C and the cooled solution was diluted with water (500 μL) and dichloromethane (1 mL). The layers were separated and the aqueous layer was extracted with dichloromethane (3 × 1 mL). The combined organic layers were washed with a saturated aqueous solution of sodium bicarbonate (3 mL), followed by a saturated aqueous solution of sodium chloride (3 mL). The washed solution was dried over magnesium sulfate, the dried solution was filtered and the filtrate was concentrated under reduced pressure at 23 °C to afford the α-bromide 15 as a pale-yellow syrup (assumed: 21.2 mg, 57.7 μmol), which was used in the next step without purification.

TLC (50% ethyl acetate in cyclohexane): Rf = 0.52 (CAM, KMnO4).

1H NMR (400 MHz, chloroform-d) δ 6.70 (d, J = 4.8 Hz, 1H), 5.80 (brs, 1H), 5.13 (d, J = 4.9 Hz, 1H), 4.36 – 4.30 (m, 1H), 2.18 (s, 3H), 2.14 (s, 3H), 1.99 (s, 3H), 1.95 (s, 3H), 1.20 (d, J = 6.5 Hz, 3H).

(3R,4S,5S,6R)-2-Fluoro-4,6-dimethyltetrahydro-2H-pyran-3,4,5-triyl triacetate (17) 

The bromide 15 (28.9 μmol scale) was prepared according to the procedure described above (see page S14) and was then dissolved in acetone (1.0 mL) and water (10 μL). The solution was cooled to 0 °C and silver carbonate (3.97 mg, 14.5 μmol, 50.0 mol%) was added at 0 °C. Upon addition, the reaction was allowed to warm up to 23 °C overnight. After 12 h, the reaction was filtered through a pad of Celite and the pad was rinsed with ethyl acetate (3 mL). The filtrate was concentrated under reduced pressure to afford the crude hemiacetal (assumed: 8.75 mg, 28.9 μmol) which was used in the next step without further purification.

Diethylaminosulfur trifluoride (DAST) (4.86 μL, 37.6 μmol, 1.30 equiv) was added dropwise to a solution of the unpurified hemiacetal (assumed: 8.75 mg, 28.9 μmol, 1 equiv) in tetrahydrofuran (100 μL) at –78 °C. Upon addition, the reaction was allowed to warm up to −40 °C over four hours. Afterwards, water (1 mL) was added at −40 °C and the resulting biphasic reaction mixture was allowed to warm up to 23 °C. The layers were separated and the aqueous layer was extracted with ethyl acetate (3 × 1 mL). The combined organic layers were washed with saturated aqueous solution of sodium chloride (2 mL). The washed solution was dried over magnesium sulfate, the dried solution was filtered and the filtrate was concentrated under reduced pressure. The crude product was purified by flash column chromatography on silica gel (10% ethyl acetate in cyclohexane, grading to 20% ethyl acetate in cyclohexane) to provide separately the pure anomers, α-glycosyl fluoride 17 (4.50 mg, 14.7 μmol, 51% over three steps) and β-glycosyl fluoride 17 as a colorless oil (900 μg, 2.90 μmol, 10% over three steps) as a colorless oil.

α-17 

TLC (50% ethyl acetate in cyclohexane): Rf = 0.50 (CAM).

1H NMR (400 MHz, chloroform-d) δ 5.80 (brs, 1H), 5.69 (dd, J = 55.3, 3.4 Hz, 1H), 5.23 (dd, J = 25.6, 3.4 Hz, 1H), 4.37 – 4.31 (m, 1H), 2.18 (s, 3H), 2.15 (s, 3H), 1.94 (s, 3H), 1.87 (s, 3H), 1.19 (d, J = 6.5 Hz, 3H).

13C NMR (151 MHz, chloroform-d) δ 170.4, 169.7, 169.3, 105.7, 104.2, 79.6, 79.6, 73.8, 70.5, 70.4, 66.9, 21.9, 21.0, 20.7, 18.0, 18.0, 16.8.

19F NMR (565 MHz, chloroform-d) δ −144.56 (dd, J = 55.2, 25.6 Hz).

[α]D20 = −1.7 (c=0.02, CH2Cl2).

IR (Diamond-ATR, neat) max: 2990 (w), 1755 (s), 1439 (w), 1373 (m), 1326 (w), 1223 (s), 1161 (m), 1130 (m), 1072 (m), 1021 (w), 932 (w), 901 (w), 868 (w), 806 (w), 753 (w), 672 (w), 643 (w), 612 (w), 482 (w) cm−1.

HRMS (ESI) calc. for C13H19FNaO7 [M+Na]+: 329.1007; found: 329.1002.

Crystal structure: see chapter 4, page S36 for more details.

β-17

TLC (50% ethyl acetate in cyclohexane): Rf = 0.43 (CAM).

1H NMR (400 MHz, chloroform-d) δ 5.69 – 5.67 (m, 1H), 5.41 (dd, J = 13.3, 7.1 Hz, 1H), 5.27 (dd, J = 53.2, 7.1 Hz, 1H), 4.06 – 4.00 (m, 1H), 2.17 (s, 3H), 2.16 (s, 3H), 1.95 (s, 3H), 1.70 (s, 3H), 1.27 (d, J = 6.4 Hz, 3H).

13C NMR (151 MHz, chloroform-d) δ 169.9, 169.4, 169.3, 107.2, 105.8, 80.6, 80.6, 73.0, 71.8, 71.6, 69.6, 69.6, 21.9, 21.0, 20.7, 17.7, 16.9.

19F NMR (377 MHz, chloroform-d) δ −142.35 (dd, J = 52.9, 13.6 Hz).

[α]D20 = +8.8 (c=0.04, CH2Cl2).

IR (Diamond-ATR, neat) max: 2990 (w), 2946 (w), 2920 (w), 1755 (s), 1443 (w), 1373 (m), 1313 (w), 1221 (s), 1175 (w), 1113 (m), 1052 (m), 957 (w), 922 (w), 889 (w), 819 (w), 718 (w), 680 (w), 594 (w), 520 (w), 485 (w), 449 (w) cm−1.

HRMS (ESI) calc. for C13H19FNaO7 [M+Na]+: 329.1007; found: 329.1002.

(2R,3S,4S,5R)-2,4-Dimethyl-6-(2,2,2-trichloro-1-iminoethoxy)tetrahydro-2H-pyran-3,4,5-triyl triacetate (18)

The bromide 15 (57.7 μmol scale) was prepared according to the procedure described above (see page S14) and was then dissolved in acetone (2.0 mL) and water (20 μL). The solution was cooled to 0 °C and silver carbonate (7.93 mg, 28.8 μmol, 50.0 mol%) was added at 0 °C. Upon addition, the reaction was allowed to warm up to 23 °C overnight. After 12 h, the reaction was filtered through a pad of Celite and the pad was rinsed with ethyl acetate (5 mL). The filtrate was concentrated under reduced pressure to afford the crude hemiacetal (assumed: 17.5 mg, 57.5 μmol) which was used in the next step without further purification.

1,8-Diazabicyclo[5.4.0]undec-7-ene (DBU) (4.31 μL, 28.9 μmol, 50.0 mol%) and trichloroacetonitrile (34.7 μL, 346 μmol, 6.00 equiv) were subsequently added to a solution of the unpurified hemiacetal (assumed: 17.5 mg, 57.5 μmol) in dichloromethane (570 μL) at 0 °C. After one hour, the ice bath was removed and water (1 mL) was added. The layers were separated and the aqueous layer was extracted with dichloromethane (3 × 1 mL). The combined organic layers were washed with saturated aqueous solution of sodium chloride (2 mL). The washed solution was dried over magnesium sulfate, the dried solution was filtered and the filtrate was concentrated under reduced pressure. The crude product was purified by flash column chromatography on silica gel (10% ethyl acetate in cyclohexane, grading to 20% ethyl acetate in cyclohexane) to provide separately the pure anomers, α-glycosyl trichloroacetimidate 18 (10.8 mg, 24.1 μmol, 42% over three steps) as a colorless oil and β-glycosyl trichloroacetimidate 18 (1.8 mg, 4.0 μmol, 7% over three steps) as a colorless oil.

α-18 

TLC (50% ethyl acetate in cyclohexane): Rf = 0.58 (CAM).

1H NMR (400 MHz, chloroform-d) δ 8.61 (s, 1H), 6.49 (d, J = 4.4 Hz, 1H), 5.80 (s, 1H), 5.43 (d, J = 4.4 Hz, 1H), 4.34 (q, J = 6.5 Hz, 1H), 2.16 (s, 3H), 2.09 (s, 3H), 1.97 – 1.93 (m, 6H), 1.18 (d, J = 6.5 Hz, 3H).

13C NMR (101 MHz, chloroform-d) δ 170.2, 169.7, 169.3, 161.3, 94.0, 91.2, 80.0, 74.0, 70.0, 67.0, 22.0, 20.8, 20.7, 17.8, 17.1.

[α]D20 = +95.8 (c=0.11, CH2Cl2).

IR (Diamond-ATR, neat) max: 3320 (w), 2990 (w), 2944 (w), 1755 (s), 1674 (m), 1555 (w), 1439 (w), 1371 (m), 1223 (s), 1128 (m), 1068 (s), 1041 (s), 969 (m), 932 (w), 901 (w), 843 (w), 796 (m), 748 (w), 641 (w), 610 (w), 487 (w), 435 (w) cm−1.

HRMS (ESI) calc. for C15H20Cl3NNaO8 [M+Na]+: 470.0147; found: 470.0145.

β-18 

TLC (50% ethyl acetate in cyclohexane): Rf = 0.46 (CAM).

1H NMR (400 MHz, chloroform-d) δ 8.64 (s, 1H), 5.87 (d, J = 8.4 Hz, 1H), 5.72 – 5.70 (m, 1H), 5.55 (d, J = 8.4 Hz, 1H), 4.08 (qd, J = 6.5, 1.3 Hz, 1H), 2.19 (s, 3H), 2.07 (s, 3H), 1.95 (s, 3H), 1.78 (s, 3H), 1.24 (d, J = 6.4 Hz, 3H).

β-18 underwent decomposition when dissolved in deuterated chloroform within two days in the NMR tube, prior to obtaining either a 13C NMR spectrum or measuring the optical rotation.

IR (Diamond-ATR, neat) max: 3447 (w), 3340 (w), 3313 (w), 2990 (w), 2934 (w), 1755 (s), 1678 (w), 1598 (w), 1441 (w), 1373 (m), 1250 (s), 1221 (s), 1140 (w), 1066 (s), 918 (w), 837 (w), 798 (w), 715 (w), 645 (w), 596 (w), 563 (w), 503 (w) cm−1.

HRMS (ESI) calc. for C15H20Cl3NNaO8 [M+Na]+: 470.0147; found: 470.0139.

# 3	NMR Spectra

 

# 4	Crystallographic Data

(2R,3R,4S,5S,6R)-2-Fluoro-4,6-dimethyltetrahydro-2H-pyran-3,4,5-triyl triacetate (α-17)

Ein Bild, das Zeichnung, Cartoon, Kunst enthält.

Automatisch generierte Beschreibung

CCDC 2337521 contains the supplementary crystallographic data for α-17

Identification code 	mar24_5

Empirical formula 	C13H19FO7

Formula weight 	306.28

Temperature 	183.00 K

Wavelength 	0.71073 Å

Crystal system 	Orthorhombic

Space group 	P212121 (no. 19)

Unit cell dimensions	a = 8.4444(3) Å	α= 90°.

	b = 12.5627(7) Å	β= 90°.

	c = 14.0772(7) Å	γ = 90°.

Volume	1493.37(12) Å3

Z	4

Density (calculated)	1.362 Mg/m3

Absorption coefficient	0.118 mm-1

F(000)	648

Crystal size	0.08 x 0.05 x 0.03 mm3

Theta range for data collection	2.173 to 25.035°.

Index ranges	−10<=h<=9, −14<=k<=14, −16<=l<=16

Reflections collected	18654

Independent reflections	2632 [R(int) = 0.0568]

Completeness to theta = 25.035°	100.0 % 

Absorption correction	Semi-empirical from equivalents

Max. and min. transmission	0.9579 and 0.8874

Refinement method	Full-matrix least-squares on F2

Data / restraints / parameters	2632 / 0 / 196

Goodness-of-fit on F2	1.034

Final R indices [I>2sigma(I)]	R1 = 0.0360, wR2 = 0.0879

R indices (all data)	R1 = 0.0445, wR2 = 0.0937

Absolute structure parameter	0.0(6)

Extinction coefficient	0.012(2)

Largest diff. peak and hole	0.127 and −0.147 e.Å-3

[^1]: W.C. Still, M. Kahn, A. J. Mitra, J Org. Chem.1978, 43, 14, 2923−2925.
[^2]: W. G. Kofron, L. M. Baclawski, J. Org. Chem. 1976, 41, 10, 1879−1880.
[^3]: C. Meisenbichler, F. Kluibenschedl, T. Müller, Anal. Chem. 2020, 92, 21, 14314–14318.
[^4]: G. M. Sheldrick, Acta Crystallogr. Sect. Found. Adv. 2015, 71, 3–8.
[^5]: G. M. Sheldrick, Acta Crystallogr. Sect. C Struct. Chem. 2015, 71, 3–8.
[^6]: N. P. Bizier, S. R. Atkins, L. C. Helland, S. F. Colvin, J. R. Twitchell, M. J. Cloninger, Carbohydr. Res. 2008, 343, 1814–1818.
[^7]: K. C. Nicolaou, C. W. Hummel, M. Nakada, K. Shibayama, E. N. Pitsinos, H. Saimoto, Y. Mizuno, K.-U. Baldenius, A. L. Smith, J. Am. Chem. Soc. 1993, 115, 7625−7635.
[^8]: G. Catelani, E. Mastrorilli, J. Chem. Soc., Perkin Trans. 1 1983, 11, 2717−2721.
[^9]: J. R. Romeo, L. McDermott, C. S. Bennett, Org. Lett. 2020, 22, 9, 3649–3654.
[^10]: D. B. Berkowitz, S. J. Danishefsky, G. K. Schulte, J. Am. Chem. Soc. 1992, 114, 4518−4529.
[^11]: J. Zeng, G. Sun, W. Yao, Y. Zhu, R. Wang, L. Cai, K. Liu, Q. Zhang, X.-W. Liu, Q. Wan, Angew. Chem. Int. Ed. 2017, 56, 5227−5231.
[^12]: C. M. Timmers, M. A. Leeuwenburgh, J. C. Verheijen, G. A. van der Marel, J. H. van Boom, Tetrahedron: Asymmetry 1996, 7, 1, 49−52.
[^13]: A. Kirschning, J. Org. Chem. 1995, 60, 5, 1228–1232.
[^14]: S. Yalamanchili, W. Miller, X. Chen, C. S. Bennett, Org. Lett. 2019, 21, 9646-9651.
[^15]: A. J. Pihko, K. C. Nicolaou, A. M. P. Koskinen, Tetrahedron: Asymmetry 2001, 12, 937–942.
